# In Vitro Inhibition of Hsp90 Protein by Benzothiazoloquinazolinequinones Is Enhanced in The Presence of Ascorbate. A Preliminary In Vivo Antiproliferative Study

**DOI:** 10.3390/molecules25040953

**Published:** 2020-02-20

**Authors:** Jaime A. Valderrama, David Ríos, Giulio G. Muccioli, Pedro Buc Calderon, Julio Benites

**Affiliations:** 1 Química y Farmacia, Facultad de Ciencias de la Salud, Universidad Arturo Prat, Casilla 121, Iquique 1100000, Chile; darios@unap.cl (D.R.); pedro.buccalderon@uclouvain.be (P.B.C.); 2 Instituto de Ciencias Exactas y Naturales, Universidad Arturo Prat, Casilla 121, Iquique 1100000, Chile; 3 Bioanalysis and Pharmacology of Bioactive Lipids (BPBL), Louvain Drug Research Institute, Université catholique de Louvain, 72 Avenue E. Mounier, BPBL 7201, 1200 Brussels, Belgium; giulio.muccioli@uclouvain.be; 4 Research Group in Metabolism and Nutrition, Louvain Drug Research Institute, Université catholique de Louvain, 73 Avenue E. Mounier, 1200 Brussels, Belgium

**Keywords:** benzothiazoloquinazoline, quinones, antiproliferative activity, clonogenic assay, Hsp90, PARP protein

## Abstract

A series of benzo[*g*]benzothiazolo[2,3-*b*]quinazoline-7,12-quinones were prepared from 2-acylnaphthohydroquinones and 2-aminobenzothiazoles and were evaluated for their in vitro antiproliferative activity. After screening using the MTT reduction assay, their IC_50_ values were calculated on a panel of cancer cells (T24, DU-145, MCF-7). Current standard anticancer drugs were included as control, and their calculated IC_50_ values were 7.8 and 23.5 µM for 5-fluorouracil and tamoxifen, respectively. Non-cancer cells (AG1523) were included to assess cancer cell sensitivity and drug selectivity. Four members of the series, with IC_50_ values from 0.11 to 2.98 µM, were chosen for further assays. The selected quinones were evaluated regarding their effects on cancer cell proliferation (clonogenic assay) and on Hsp90 and poly(ADPribose)polymerase (PARP) protein integrity. The most active compound (i.e., **15**) substantially inhibited colony forming unit (CFU) formation at 0.25 µM. In the presence of ascorbate, it induced an oxidative cleavage of Hsp90 but had no effect on PARP protein integrity. In an in vivo animal model, it discreetly increased the mean survival time (m.s.t.) of tumor-bearing mice. In light of these results, compound **15** represents a potential lead-molecule to be further developed.

## 1. Introduction

Many natural and synthetic compounds sharing the 1,4-naphthoquinone scaffold display a wide variety of biological activities [1]. A key mechanism explaining the activities of such molecules is the intercalation of DNA due to the high ability of their large planar polycycles to bind the base pairs through hydrogen bonds and “π-stacking” interactions [2,3]. Hence, an optimal DNA intercalation occurs with compounds containing three to four coplanar rings, such as mitoxantrone and doxorubicin. This interferes with normal DNA functioning and leads to cell death [4]. Interestingly, depending on the molecules, such DNA damage can be caused by the parent form or following its metabolic conversion to electrophilic or radical species [5]. In this context, quinones having redox-cycling properties are endowed with potential anticancer activities [1,2,3,4,5,6,7,8,9]. The rationale behind this is based on a particular ambivalence of cancer cells: they produce a large amount of reactive oxygen species (ROS), while they are generally deficient in antioxidant enzymes [10,11,12,13]. Such a dichotomy represents a vulnerability of tumor cells to an oxidative stress, which can be therapeutically exploited. Indeed, an ROS-generating system (i.e., quinone redox cycling) yields a huge amount of ROS that exceeds the antioxidant defense capacity, thus, compromising their fine redox equilibrium. In this context, we have induced the alteration of intracellular redox homeostasis of cancer cells by using redox-cycler quinones as a new strategy in the research and development of new antitumor drugs. To this end, numerous quinone derivatives have been synthesized and assessed for their biological activity in order to optimize this redox-cycling approach [11,14,15,16].

Recently, we have reported results on the synthesis and preliminary in vitro cytotoxic evaluation of a coplanar heteroaromatic scaffold namely benzo[*g*]benzothiazolo[2,3-*b*]quinazoline-7,12-quinones [16]. The synthetic accessibility to these heterocycles as well as the encouraging cytotoxic activities of some members of the series on cancer cells suggest that these heterocyclic quinones might be good antitumor compound candidates. Based on these preliminary results, we assessed the anticancer potential of the previously reported benzothiazoloquinazolinequinones [16] together with new members of the series using a double in vitro/in vivo approach. The in vitro effect of quinones was compared to two well-known anticancer drugs, namely tamoxifen (TAM) and 5-fluorouracil (5-FU). TAM has been utilized against prostate and mammary cancer cells [17], while 5-FU has been used against bladder tumors [18,19]. Moreover, to bring some mechanistic insight to the mechanism of action, we have also evaluated the effect of quinones on important intracellular cancer cell targets, namely poly(ADPribose)polymerase (PARP) and the chaperone Hsp90 protein. Previous studies have shown that his latter protein is a good target for oxidant-based antitumor treatments [20]. 

## 2. Results

### 2.1. Synthesis of Benzo[G]Benzothiazolo[2,3-B]Quinazoline-7,12-Quinones

The quinones employed in this study were prepared by heteroannulation between acylnaphthoquinones generated in situ from their respective naphthohydroquinones **1**–**6**, and 2-aminobenzothiazoles **7**–**10** according to our previously reported procedure [16], (Supplementary data). Table 1 summarizes the results for the preparation of the members of the series **11**–**26**. The structures of quinones **11**–**16**, **18**, **19**, **21**, **22** were confirmed by comparing their spectral properties (^1^H, ^13^C NMR) to those reported in the literature [16]. According to the proposed mechanism for heterocycle heteroannulation [16], the reaction is initiated by attacking the NH_2_ group of the aminobenzothiazoles at the 3-position of the acylnaphthoquinones. This attack generates Michael intermediate adducts, which by a further 6-exo trig ring closure followed by aerobic oxidation yields the heterocycles containing a chiral carbon atom at the 13-position of the heteropentacyclic scaffold.

### 2.2. In Vitro Antitumor Activity of Heterocyclic Quinones

As a first approach, an MTT reduction test was performed using a panel of human cancer (MCF-7, T24, and DU-145), non-tumor (AG1523) cell lines in the presence of increasing concentrations of the test compounds. As positive control, two well-known anticancer drugs, namely TAM and 5-FU, were also tested on these cell lines. TAM, a non-steroidal anti-estrogen drug, is of interest because it has been used against both MCF-7 and DU-145 cells [17]. Regarding 5-FU, it is a broad-spectrum chemotherapeutic drug used to treat a variety of malignancies. Particularly, it has a long history of use against bladder tumors [18,19]. 

Table 2 shows that the most active quinones were compounds **15**, **16**, **19**, and **20** with IC_50_ values ranging from 0.11 to 1.77 µM. Excluding these compounds, no particular sensitivity of cancer cell lines was observed with regard to quinones although DU-145 cells seemed a little more resistant compared to both MCF-7 and T24 cells. Otherwise, a more dispersed range of sensitivity was noticed within the quinone series in healthy fibroblasts.

By taking a mean IC_50_ value calculated from the individual IC_50_ values calculated for the three cancer cell lines, the quinones may be ranked in three groups with different activities. The first one, encompassing the most active quinones, included compounds **15**, **16**, **19**, and **20** (mean IC_50_ values from 1.10 to 1.61 µM). A second group is shown by the less active quinones (**23** and **26**) with IC_50_ mean values ranging from 10.52 to 12.61 µM. The third one included quinones with IC_50_ values ranging from 5.36 to 8.92 µM. It should be noted that 12 out of 16 quinones were more active than 5-fluorouracil. Finally, all quinones had IC_50_ values lower than those obtained with TAM. 

Table 2 also shows the mean selectivity index (MSI) of quinones and the standard drugs. Such a selective index was calculated as the ratio between IC_50_ values of healthy cells/IC_50_ values in cancer cells. Interestingly, the most active quinones, namely **15**, **16**, **19**, and **20**, as well as a less active compound **24** show high values of MSI. 

The data in Table 2 can be used to establish a preliminary structure–activity relationship (SAR) analysis. Such SAR study reveals that for quinones **11**–**14** and **23**–**26**, the nature and size of the carbon ligands bonded to the chiral carbon atom of the pentacyclic scaffold did not significantly influence either their activity or their selectivity. The substitution effects are more significant in enhancing the antiproliferative activity for the members containing the methyl or propyl groups in the 13-position. Thus, the insertion of methyl or methoxy groups in the 3-position of the compounds **11** and **12**, as in **15**, **16**, **19**, and **20**, induces a dramatic increase of the antiproliferative activity compared to those of their quinone precursors, reaching in some cases nanomolar IC_50_ values.

Based on these results, the most active quinones, namely **15**, **16**, **19**, and **20**, were chosen for a clonogenic assay. Table 3 shows the effects of four selected quinones on the growth capacity of T24 cells as shown by the number of colony forming units (CFUs). Compound **15** appeared as the most active member of the series. Indeed, at a dose of 0.25 µM, it decreased from 195 to 72 the number of CFUs. Otherwise, it inhibits the proliferation of T24 cells by 63% as compared to the control untreated cells. At such concentration, the other compounds showed a residual activity with the exception of **19**, which is able to inhibit cell proliferation by 28%, however, it is still two times less active than **15**. The percentages of the observed cell growth compared to control conditions (100%) are indicated in brackets.

### 2.3. Effect of Selected Quinones on Intracellular Targets

Next, we wanted to assess how these quinone derivatives affect cancer-related intracellular targets. Here, we selected Hsp90 and PARP. Indeed, Hsp90 is highly overexpressed in cancer cells, where it plays a critical role in stabilizing proteins that are essential for carcinogenesis such as Akt, RIP, and others [20]. PARP, and more specifically PARP cleavage, is a classical marker of apoptosis making it a relevant target to assess here.

**Figure 1:**
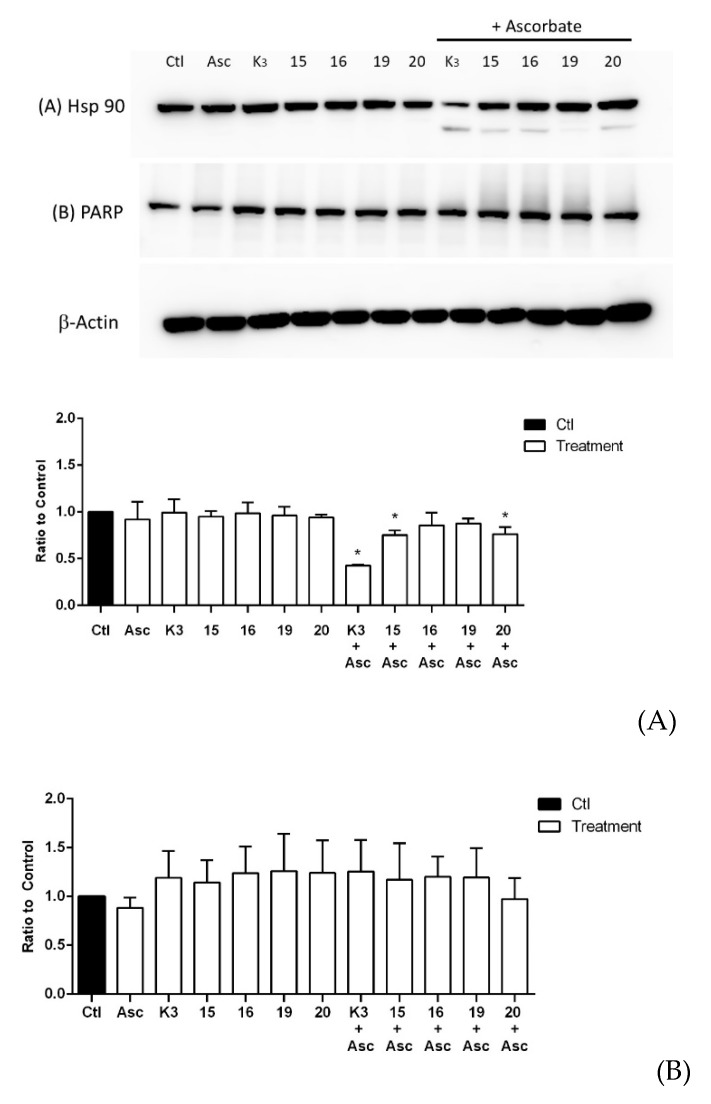
Effects of selected quinones of Hsp90 (**A**) and poly(ADPribose)polymerase (PARP) (**B**) protein integrity in T24 cells. Cancer cells were incubated for 4 h with 5 µM menadione (K3), **15, 16, 19**, or **20** either in the absence or in the presence of 1 mM ascorbate (Asc). Ctl represents control untreated cells. * *p* < 0.05 as compared to control conditions.

Figure 1A shows the effect of four selected quinones on a chaperone Hsp90 protein. When T24 cells were incubated with quinones (5 µM), no changes were observed with regard to the integrity of the Hsp90 protein. Nevertheless, when incubation was done in the presence of 1 mM ascorbate (vitamin C) plus quinones (including menadione), a new peptide fragment of about 70 kD was detected by the antibody against Hsp90. In previous studies, it was demonstrated that ascorbate/menadione (asc/men) provoked an oxidative proteolytic cleavage at the N-terminal domain of Hsp90, and the new peptide fragment did not come from a new protein synthesis [17,21,22]. The results depicted in Figure 1A show that all quinones (**19** to a lesser extent) are engaged within a redox cycle induced by ascorbate generating ROS, which ultimately leads to Hsp90 protein cleavage and likely to a loss of its chaperone function. Notably, the incubation of ascorbate with menadione induced a strong Hsp90 cleavage as compared to synthesized quinones.

Regarding PARP cleavage, neither the quinone derivatives nor menadione induced a detectable PARP cleavage (Figure 1B). This was the case regardless of the presence of ascorbate (1 mM). The obtained results make a potential role of apoptosis unlikely to be the underlying mechanism of cell impairment induced by quinones, including menadione. Indeed, PARP is a substrate of caspase-3, therefore, when an apoptosis process is triggered, it is expected that PARP protein will be cleaved yielding a second protein band also recognized by the anti-PARP antibody. 

**Figure 2:**
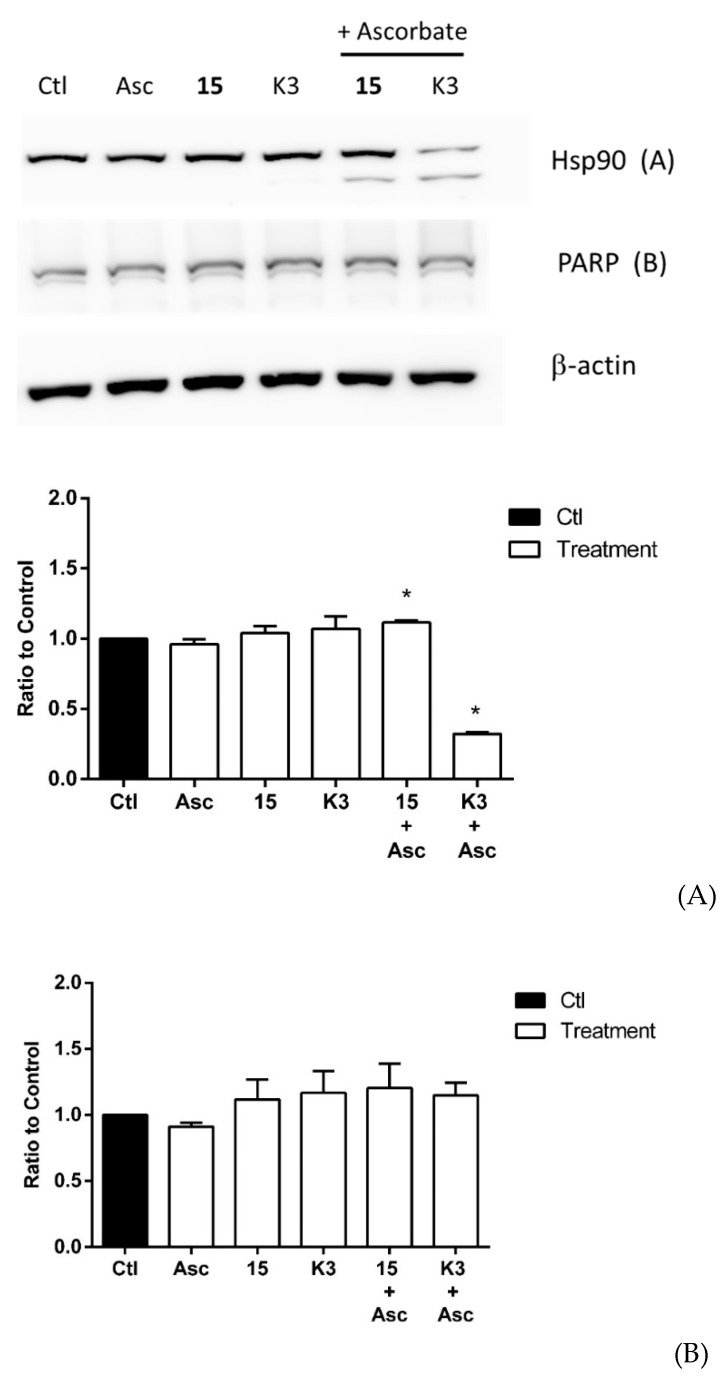
Effects of selected quinones of hsp90 (**A**) and PARP (**B**) protein integrity in transplantable primary liver tumor (TLT) cells. Cancer cells were incubated for 4 h with 5 µM menadione (K3) or **15** either in the absence or in the presence of 1 mM ascorbate (Asc). Ctl represents control untreated cells. * *p* < 0.05 as compared to control conditions.

Figure 2 shows the effects of quinone **15** on Hsp90 (Figure 2A) and PARP protein (Figure 2B) by using the hepatocarcinoma TLT cells instead of T24 cells. As already observed by using the T24 cell line, quinones in the presence of ascorbate were able to produce the same effect on TLT cells: oxidative cleavage of Hsp90 but not PARP degradation.

### 2.4. In Vivo Antitumor Activity by ***15***

**Figure 3:**
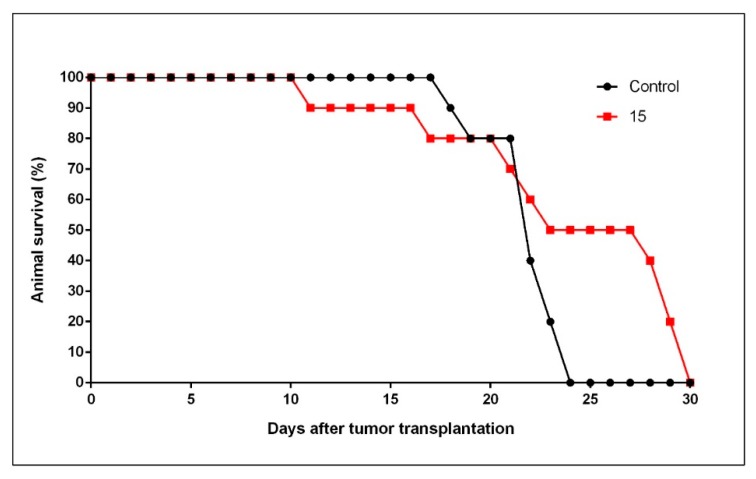
Kaplan–Meier diagram in groups of TLT-bearing NMRI mice receiving intraperitoneal (i.p.) saline (control untreated animals). Compound **15** was also i.p. administered as 10 mg/kg body weight, 24 h after i.p. transplantation of TLT cells.

To go one-step further, we assessed the effect of the quinone derivative **15** in the TLT-bearing mice model. The dose of **15** used in this study (i.e., 10 mg/kg) was calculated according to doses of menadione, previously used by Verrax et al. [23]. In mice treated with **15,** the calculated mean survival time was 24.0 days as compared to 21.9 days in control untreated-animals, representing an increase in life span of 9.6 % (Figure 3). Although this ILS represents a marginal effect as compared to a minimum optimal effect (<25%), a small trend can be inferred supporting a potential protective activity by **15**, that requires to be further confirmed. When considering how long every individual mouse survived, a mean of 22.8 ± 6.1 days was obtained for the experimental treated group while the control group had a mean of 21.2 ± 1.6 days. A two-sample t-test on survival time (in days) revealed no significant difference between treated and control group (t(38) = 1.13, *p* = 0.262). To go further, studies are intended in the near future using a larger range of doses of **15**.

## 3. Materials and Methods

### 3.1. Synthesis Of Benzo[G]Benzothiazolo[2,3-B]Quinazoline-7,12-Quinones

The new members of the series **17**, **20**, **23**–**26** were prepared by heteroannulation between acylnaphthoquinones in situ generated from their respective naphthohydroquinones **2**, **3**, **5**, **6**, and 2-aminobenzothiazoles **7**–**10** according to our previously reported procedure [16]. NMR data for compound **23** is not reported due to its extremely low solubility in the common solvents used in NMR spectroscopy (the experimental procedure and the characterization of the compounds is reported in the Supplementary Materials). 

### 3.2. Cell Lines and Cell Cultures

Human cancer cell lines T24 (bladder), DU-145 (prostate), MCF-7 (breast) and non-tumor fibroblasts AG 1523 were obtained from the American Type Culture Collection (ATCC, Manassas, VA, USA). Cell culture conditions were the same as reported elsewhere [18]. Briefly, cell lines were kept in DMEM containing 10% fetal calf serum. In addition, 100 U/mL of penicillin and 100 µg/mL streptomycin from Gibco (Grand Island, NY, USA) were further added. Cell cultures were kept at 37 °C under an atmosphere of 95% air/5% CO_2_ and 100% humidity. Sodium L-ascorbate, menadione (vitamin K3), TAM, and 5-FU were purchased from Sigma (St. Louis, MO, USA).

### 3.3. Cell Survival Assays 

The cytotoxicity of the quinones was assessed by following the reduction of MTT (3-(4,5-dimethylthiazol-2-yl)-2,5-diphenyltetrazolium bromide) to formazan blue [24] and the capability of cancer cells to proliferate, namely a clonogenic assay [25]. 

#### 3.3.1. MTT Reduction Assay 

Cells were seeded into 96-well plates at a density of 10,000 cells/well for 24 h and then incubated for 48 h with or without the quinone derivatives. Tamoxifen and 5-fluorouracil were used as standard chemotherapeutic agents (positive controls). The IC_50_ values were determined using the GraphPad Prism software (San Diego, CA, USA). Further incubation procedures and optical density reading of colored solutions were performed according to Benites et al. [26].

#### 3.3.2. Clonogenic Assays 

They were performed by seeding T24 cells (500) in six-well plates at a single-cell density. T24 cells were chosen because of their facility to enumerate when they form colonies while the other cell lines form cellular aggregates during their proliferation making the counting process difficult. Cells were allowed to adhere overnight, and then treated with quinones for 24 h. Afterward, they were washed with warm PBS, given fresh medium, and allowed to grow for 10 days. Clonogenic survival was determined by fixing and staining colonies using crystal violet and further counting their number. The number of colonies calculated under control conditions was set as 100%.

### 3.4. Immunoblotting Procedures 

Cancer cells were incubated for 4 h in the absence and in the presence of compounds. Afterward, cells were washed twice with ice-cold PBS and then resuspended in RIPA lysis buffer supplemented with 1% protease inhibitor cocktail (Sigma–Aldrich, St. Louis, MO, USA) and 3% phosphatase inhibitor cocktail (Calbiochem, Darmstadt, Germany). Treatment of samples and electrophoresis conditions were conducted as previously reported [24]. Mouse monoclonal primary antibodies against hsp90α/β C-terminus (F8), and against β-actin (clone AC-15), were purchased from Santa Cruz Biotechnology (Santa Cruz, CA, USA) and from Abcam (Cambridge, UK), respectively. Rabbit polyclonal antibodies against PARP were purchased from BD Biosciences (San Diego, CA, USA). Goat anti-rabbit antibody and rabbit anti-mouse polyclonal antibody were purchased respectively from Chemicon International (Temecula, CA, USA) and DakoCytomation (Glostrup, Denmark).

### 3.5. Animals and Diet 

Young adult male NMRI mice (25–30 g) were obtained from Janvier Labs (Saint-Berthevin, France). After their placement at the University Animal Facilities, animals were randomized according to their body weight. They were housed in groups of five mice per cage (40 × 25 × 15 cm^3^) in a constant temperature environment (22 °C) with alternating 12 h day/night cycles receiving standard food (UAR, Villemoisson-sur-Orge, France) and tap water ad libitum. 

### 3.6. Experimental Tumor Model 

Taper et al. [27] first observed the transplantable primary liver tumor (TLT) in 1966 in a two-month old female Swiss Webster mouse. It had a rapid growth in both solid and ascetic forms and it was called hepatoma indicating the organ of its origin. Fritzler et al. [28] further characterized the ultrastructure of TLT hepatoma ascites cells. Regarding our experimental conditions, the TLT tumor was maintained by weekly intraperitoneal (i.p.) transplantation (10^4^ cells/mice). For antitumor assays, 1 × 10^6^ TLT cells were i.p. implanted in NMRI mice, and after 24 h, animals were i.p. receiving a solution of either saline (control group) or **15** (10 mg/kg body weight). After intraperitoneal tumor transplantation, animal health conditions were daily verified. To avoid unnecessary animal suffering, mice were killed by cervical dislocation when posterior leg paralysis was noted. Then, mice mortality was daily recorded. Such a protocol was repeated twice, with a final number of 20 mice per experimental group. Procedures were conducted in accordance with legal requirements and with the approval of the local bioethics committee (2014/UCL/MD/010). 

The antitumor activity was calculated according to Geran et al. [29] using the mean survival time (m.s.t.) at day 30 and the increase in life span (ILS) calculated as ILS = [(m.s.t. control/m.s.t. 15) − 1] × 100. This murine hepatoma-derived cell line was selected for the in vivo experiments because of its previous use for oncologic studies [30,31] and due to its deficiency in antioxidant enzymes [32].

### 3.7. Statistical Analysis 

All in vitro experiments were done at least three times. The experimental data were examined using either a one-way ANOVA or an unpaired t-test, using GraphPad Prism software (GraphPad Software, San Diego, CA, USA). A value of *P* < 0.05 was set as the level of significance. The statistical analyses of the treatment of mice were done according to procedures reported by Geran et al. [29].

## 4. Discussion

Severe adverse effects frequently restrain the successful achievement of antitumor chemotherapy. Due to such current difficulties, the search for new drugs and alternative therapies has become a crucial issue. In this context, we have developed a new and original series of quinones endowed with in vitro antiproliferative activity in the range of 0.1–20 µM, as shown by the MTT reduction test and a clonogenic assay. The molecular mechanisms by which quinones display their anticancer activities remain elusive, but they are likely different from those of both TAM and 5-FU. Indeed, in vitro studies indicate that TAM is a potent inhibitor of PKC [33,34], while the mechanism of cytotoxicity of 5-FU has been attributed to fluoronucleotide misincorporation into DNA inhibiting DNA synthesis thus leading to cell death [35]. Despite these differences in the mechanism of action, the range of in vitro activities by quinones was in the same order of magnitude of standard anticancer drugs [17]. Interestingly, when some of these quinones were compared with free-radical-generating anticancer drugs such as doxorubicin and mitomycin C, they were less active than doxorubicin but fairly more active than mitomycin C [16].

Currently, the best and most definitive therapeutic outcome for a potential anticancer drug is done by an in vivo assay showing tumor inhibition or at least, a delay in tumor growth. However, several issues, such as the nature of the tumor, the administration route, the doses employed, the applied schedule (single or multiple doses of the anticancer compound), etc., restrict the in vivo efficacy of a given drug. We would like to stress the point that our experimental approach implies stringent conditions, because the compound was administered as a single dose. In addition, the TLT hepatocarcinoma is extremely aggressive and no standard drugs have been capable of inhibiting in vivo TLT growth [30]. For all these reasons, it should be kept in mind that although the in vivo effect of **15** was rather small, such a low activity may represent a promising in vivo anticancer effect.

Since menadione (vitamin K3) also has the 1,4-naphthoquinone scaffold, we inferred that the selected quinones would be have a redox behavior similar to that of menadione. That means the occurrence of an ascorbate-driven quinone redox cycling leading to ROS formation that ultimately causes an oxidative Hsp90 cleavage. Indeed, we have recently shown the in situ formation of hydroxyl free radicals by a Fenton-type reaction at the N-terminal nucleotide binding pocket of Hsp90 forming a protein radical, which, by rearrangement, causes the rupture of the peptide backbone [21]. It is worth recalling that Hsp90 is a chaperone protein that stabilizes numerous proteins considered crucial for cancer cell survival. Such proteins have been called “client” proteins and the list includes Akt, RIP, Bcr-Abl (specifically in K562 cells), etc. Therefore, when Hsp90 is either destabilized due to the action of geldanamycin [4,36,37] or cleaved by an oxidative attack [20,21], its chaperone function is lost, client proteins are then degraded in the proteasome disturbing cellular equilibrium and ultimately causing cancer cell death. 

Regarding PARP protein, the obtained results are in agreement with previous reports acquired with other types of cancer cells (i.e., K562, TLT). Under such conditions, the cell demise induced by ascorbate/menadione was not caspase-3 dependent but close to a necrotic-like cell death [23,32]. Therefore, since PARP is a substrate of caspase-3, it would be expected that PARP protein should be cleaved when apoptosis is activated, which was not the case here. Although our results lead to the conclusion that apoptosis is unlikely to be playing a role, additional assays are required to better characterize the type of cell death induced by quinones. Indeed, we are working to identify which intracellular targets are impaired in order to explain the antiproliferative activity of quinones.

In conclusion, original quinones were prepared by in situ heteroannulation between acyl-naphthoquinones and substituted 2-aminobenzothiazoles. Most quinone derivatives have shown anticancer activities at lower doses than 5-FU and TAM. Four molecules (**15**, **16**, **19**, **20**) were extremely active against the three cancer cell lines. Among them, compound **15** displays a strong in vitro antiproliferative effect but a tiny in vivo activity.

## Figures and Tables

**Table 1:**
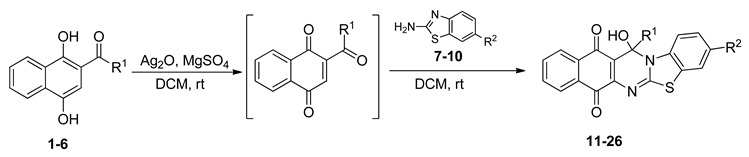
Synthesized dibenzothiazoloquinazoline-7,12-quinones **11**–**26**.

Acylhydroquinone	Aminobenzothiazol	Product	R^1^	R^2^	Yield (%) ^a^
**1**	**7**	**11**	Me	H	67 ^b^
**2**	**7**	**12**	1-Propyl	H	78 ^b^
**3**	**7**	**13**	1-Pentyl	H	47 ^b^
**4**	**7**	**14**	1-Heptyl	H	41 ^b^
**1**	**8**	**15**	Me	Me	61 ^b^
**2**	**8**	**16**	1-Propyl	Me	68 ^b^
**3**	**8**	**17**	1-Pentyl	Me	87
**4**	**8**	**18**	1-Heptyl	Me	84 ^b^
**1**	**9**	**19**	Me	OMe	43 ^b^
**2**	**9**	**20**	1-Propyl	OMe	61
**3**	**9**	**21**	1-Pentyl	OMe	75 ^b^
**4**	**9**	**22**	1-Heptyl	OMe	60 ^b^
**5**	**7**	**23**	2-Furyl	H	55
**5**	**9**	**24**	2-Furyl	OMe	63
**6**	**7**	**25**	2- Thiophen	H	63
**6**	**10**	**26**	2- Thiophen	F	63

^a^ Isolated yield after column chromatography with reference to their precursors **1**–**6**; ^b^ Reference [20].

**Table 2:** In vitro inhibitory effect of compounds **11**–**26** on the proliferation of the human-derived tumor cell lines: T24 (bladder), DU-145 (prostate), and MCF7 (breast) and the non-tumor fibroblasts (AG 1523).

IC_50_ ± SEM (Standard Error of the Mean) ^a^ (μM)						
Structure	No.	T24	DU-145	MCF-7	MSI^b^	AG 1523
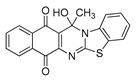	11	4.65 ± 0.43	9.59 ± 0.32	6.83 ± 1.29	1.52	10.71 ± 0.57
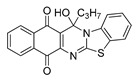	12	4.86 ± 0.50^c^	11.58 ± 2.05^c^	4.25 ± 0.50^c^	1.28	8.82 ± 0.27
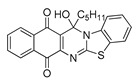	13	6.85 ± 0.54	7.99 ± 0.17	5.83 ± 0.25	1.24	8.55 ± 0.35
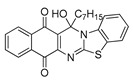	14	8.42 ± 1.27	9.04 ± 0.69	6.84 ± 0.79	0.90	7.28 ± 0.39
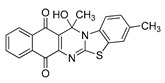	15	0.22 ± 0.06 ^c^	0.11 ± 0.03 ^c^	2.98 ± 0.52 ^c^	29.4	32.31 ± 3.31
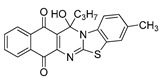	16	1.36 ± 0.15^c^	0.85 ± 0.10^c^	1.77 ± 0.26^c^	2.62	3.46 ± 0.08
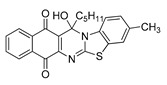	17	6.86 ± 0.33	8.46 ± 0.31	5.26 ± 0.22	1.09	7.50 ± 0.69
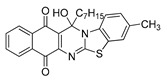	18	5.60 ± 0.29	7.55 ± 0.69	7.12 ± 0.65	0.89	6.02 ± 0.29
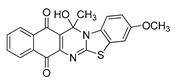	19	1.32 ± 0.11^c^	0.79 ± 0.11^c^	2.72 ± 0.18^c^	3.97	6.40 ± 0.46
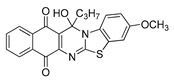	20	1.89 ± 0.15	1.13 ± 0.05	1.67 ± 0.12	3.17	4.99 ± 0.59
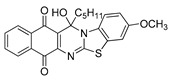	21	7.09 ± 0.14	7.43 ± 0.28	4.63 ± 0.30	1.78	11.32 ± 1.10
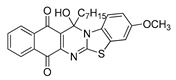	22	5.95 ± 0.45	6.62 ± 0.19	3.52 ± 0.32	1.11	5.97 ± 0.24
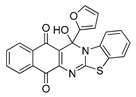	23	9.29 ± 0.77	19.95 ± 0.50	8.62 ± 0.72	1.72	21.70 ± 0.27
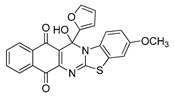	24	10.41 ± 1.00	4.69 ± 0.33	11.69 ± 0.65	10.1	90.12 ± 1.21
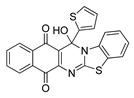	25	8.72 ± 0.41	7.85 ± 0.60	7.32 ± 0.36	1.60	12.77 ± 0.31
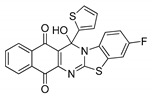	26	13.37 ± 2.37	10.75 ± 1.01	7.46 ± 0.53	1.88	19.79 ± 0.83
5-Fluorouracil	-	3.61 ± 0.77	7.10 ± 1.45	12.68 ± 1.9	0.84	6.59 ± 1.04
Tamoxifen	-	27.9 ± 4.8	23.0 ± 4.1	19.6 ± 2.6	0.95	22.5 ± 2.3

^a^ Data represent IC_50_ mean values ± SEM of at least three different experiments; ^b^ MSI: mean selective index = IC_50_ values of fibroblasts/IC_50_ values of tumor cells; ^c^ Data were got from Valderrama et al. [16].

**Table 3:** Effect by quinones on antiproliferative capacity (CFUs—colony forming units) of T24 cells.

Dose (µM)	15	16	19	20
0.00	195 ± 16 (100)	202 ± 28 (100)	191 ± 24 (100)	213 ± 23 (100)
0.025	180 ± 11 (92)	204 ± 19 (101)	195 ± 16 (102)	204 ± 17 (96)
0.125	162 ± 18 (83)	216 ± 29 (107)	183 ± 21 (96)	214 ± 25 (100)
0.25	72 ± 5 * (37)	189 ± 12 (94)	138 ± 18 * (72)	225 ± 19 (106)
1.25	22 ± 9 * (11)	79 ± 9 * (39)	68 ± 12 * (36)	118 ± 22 * (55)
2.5	16 ± 3 * (8)	58 ± 12 * (29)	30 ± 16 * (16)	89 ± 10 * (42)
5.0	2 ± 1 * (1)	23 ± 15 * (11)	35 ± 11 * (18)	46 ± 7 * (22)

T24 cells were incubated for 24 h in the absence or in the presence of varied concentrations (from 0 to 5 µM) of quinones, and the proliferative capacity of cells was evaluated as shown in the Materials and Methods section. * *p* < 0.05 as compared to control values.

## Supplementary Materials

Description of the general procedure for the preparation of benzo[*g*]benzothiazolo[2,3-*b*]quinazoline-7,12-quinones **17**, **20**, **23**–**26**, and spectral data of nuclear magnetic resonance (^1^H NMR and ^13^C NMR) and high-resolution mass spectrometry (HRMS).
