# Supplementary material for: In Vitro Inhibition of Hsp90 Protein by Benzothiazoloquinazolinequinones Is Enhanced in The Presence of Ascorbate. A Preliminary In Vivo Antiproliferative Study
Source: Molecules. 2020 Feb 20;25(4):953. doi: 10.3390/molecules25040953 (PMC7071032; doi:10.3390/molecules25040953)
Supplement: Supplementary file 1 [file molecules-25-00953-s001.pdf]

Supplementary Data

1. General Information

All the solvents and reagents were purchased from different companies such as Aldrich (St. Louis, MO, USA) and Merck (Darmstadt, Germany) and were used as supplied. Melting points were determined on a Stuart Scientific SMP3 (Staffordshire, UK) apparatus and are uncorrected. The IR spectra were recorded on an FT IR Bruker spectrophotometer, model Vector 22 (Bruker, Rheinstetten, Germany), using KBr disks, and the wave numbers are given in cm^{-1}. ^{1}H- and ^{13}C-NMR spectra were recorded on Bruker Avance-400 instrument (Bruker, Ettlingen, Germany) in $CDCl_3$ or DMSO-d_6 at 400 and 100 MHz, respectively Chemical shifts are expressed in ppm downfield relative to tetramethylsilane and the coupling constants (*J*) are reported in Hertz. Data for ^{1}H-NMR spectra are reported as follows: s = singlet, br s = broad singlet, d = doublet, m = multiplet and the coupling constants (*J*) in Hz. Bidimensional NMR techniques and distortionless enhancement by polarisation transfer (DEPT) were used for signal assignment. Chemical shifts are expressed in ppm downfield relative to tetramethylsilane and the coupling constants (*J*) are reported in Hertz. The HRMS data for all final compounds were obtained using a LTQ-Orbitrap mass spectrometer (Thermo-Fisher Scientific, Waltham, MA 02454, USA) with the analysis performed using an atmospheric-pressure chemical ionization (APCI) source operated in positive mode. Silica gel Merck 60 (70–230 mesh, from Merck) was used for preparative column chromatography and TLC aluminum foil 60F_{254} for analytical thin layer chromatography (TLC).

2. Synthesis of benzo[g]benzothiazolo[2,3-b]quinazoline-7,12-quinones

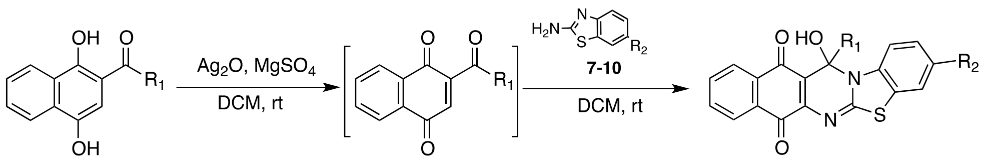

1-6 **11-26**

General Procedure. Suspensions of acylhydroquinones (**1-6;** 1 mmol), Ag_2O (*ca.* 2.5 mmol), anhydrous $MgSO_4$ (*ca.* 200 mg), and dichloromethane (10 mL) were left at room temperature with stirring at room temperature for 1 h. The mixtures were filtered, the solids were washed with dichloromethane (5 mL) and, over the filtrates, 2-aminobenzothiazoles (**7-10**; 1.1 mmol) were added, and the resulting solutions were left with stirring at room temperature for 24 h. The solvents were removed under reduced pressure and the residues were chromatographed over silica gel (petroleum ether/CH_2Cl_2/EtOAc, 15:5:20) to yield pure compounds **11-26**.

3. *Data of the new members of the series* (**17**, **20**, **24-26**)

3.1. *13-Hydroxy-13-(1-pentyl)-7H-benzo[g]benzo[4,5]-3-methylthiazolo[2,3-b]quinazolin-7,12-quinone* (**17**): was isolated (157.3 mg; 87%) as dark red solid, m.p. 129-129.5 °C from 2-hexanoyl-1,4-

naphthohydroquinone **3** (200 mg; 0.77 mmol), Ag_2O (446.1 mg; 1.9 mmol), anhydrous $MgSO_4$ (200 mg), 2-amino-6-methylbenzothiazole **8** (133.0 mg; 0.81 mmol) and CH_2Cl_2 (15 mL). Eluent mixture: (32:8 petroleum ether/EtOAc). IR (KBr, cm^{-1}): 3395 (OH); 1678 and 1640 (C=O); ^1H NMR (400 MHz, $CDCl_3$): δ 0.68 (bs, 3H, 5`-$CH_3$), 0.89 (m, 2H, 4`-CH_2), 1.08 (bs, 4H, 2`-$CH_2$ + 3`-CH_2), 2.22 (m, 1H, 1`-C<u>H</u>H), 2.65 (m, 1H, 1`-CH<u>H</u>), 5.45 (bs, 1H, 13-OH), 7.09 (d, 1H, *J* =7.9 Hz, 1-H or 2-H), 7.20 (d, 1H, *J* = 7.9 Hz, 2-H or 1-H), 7.35 (bs, 1H, 4-H), 7.71 (m, 2H, 8-H or 11-H), 8.08 (d, 1H, *J* = 7.7 Hz, 9-H or 10-H), 8.16 (d, 1H, *J* = 7.2 Hz, 10-H or 9-H); ^{13}C NMR (101 MHz, $CDCl_3$): δ 13.9, 21.3, 22.4, 24.3, 31.3, 39.6, 91.4, 114.4, 117.4, 118.8, 121.1, 122.4, 124.8, 126.0, 127.3, 128.4, 131.3, 132.3, 133.0, 133.4, 134.3, 146.5, 167.1, 182.4, 185.2; HRMS (M^+): m/z Calc. for $C_{25}H_{23}NO_3S$: 417.13986; found: 417.14086.

3.2. *13-Hydroxy-13-(1-propyl)-7H-benzo[g]benzo[4,5]-3-methoxythiazolo[2,3-b]quinazolin-7,12-quinone* (**20**): was isolated (122.0 mg; 61%) as red solid, m.p. 188.5-189.0 °C from 2-butanoil-1,4-naphthohydroquinone **2** (200 mg; 0.49 mmol), Ag_2O (283.8 mg; 1.2 mmol), anhydrous $MgSO_4$ (300 mg), 2-amino-6-methoxybenzothiazole **9** (91.9 mg; 0.51 mmol) and CH_2Cl_2 (15 mL). Eluent mixture: (34:6 petroleum ether/EtOAc). IR (KBr, cm^{-1}): 3423 (OH); 1681 and 1639 (C=O); ^1H NMR (400 MHz, DMSO-d_6): δ 0.70 (m, 3H, 3`-$CH_3$); 2.50 (m, 2H, 2`-CH_2); 3.32 (m, 2H, 1`-CH_2); 3.81 (m, 3H, 3-OCH_3); 7.08 (m, 1H, 13-OH); 7.57 (m, 1H, 4-H); 7.77 (m, 1H, 2-H); 7.80 (m, 1H, 9-H or 10-H); 7.87 (m, 1H, 10-H or 9-H); 8.01 (m, 3H, 1-H + 8-H + 11-H); ^{13}C NMR (100 MHz, DMSO-d_6): δ 14.0; 18.1; 38.1; 56.2; 91.0; 107.9; 114.9; 116.4; 118.3; 125.6; 126.1; 126.2; 131.1; 131.2; 133.4; 133.5; 134.9; 145.9; 156.9; 164.92; 181.7; 182.4; HRMS (M^+): m/z Calc. for $C_{22}H_{18}N_2O_4S$: 406.09873; found: 406.10450.

3.3. *13-Hydroxy-13-(2-furyl)-7H-benzo[g]benzo[4,5]-3-methoxythiazolo[2,3-b]quinazolin-7,12-quinone* (**24**): was isolated (43.4 mg; 63%) as dark red solid, m.p. 261-262 °C from 2-furoyl-1,4-naphthohydroquinone **5** (39.9 mg; 0,16 mmol), Ag_2O (183 mg; 0.79 mmol), anhydrous $MgSO_4$ (300 mg), 2-amino-6-methoxybenzothiazole **9** (32.4 mg; 0.18 mmol) and CH_2Cl_2 (15 mL). Eluent mixture: (10:25:5 petroleum ether/CH_2Cl_2/EtOAc). IR (KBr, cm^{-1}): 3448 (O-H); 1678 and 1654 (C=O); HRMS (M^+): m/z Calc. for $C_{23}H_{14}N_2O_5S$: 430.06234; found: 430.06258.

3.4. 13-Hydroxy-13-(2-thienyl)-7H-benzo[g]benzo[4,5]-thiazolo[2,3-b]quinazolin-7,12-quinone (**25**): was isolated (97 mg; 63%) as red solid, m.p. 229-230 °C from 2-thienoyl-1,4-naphthohydroquinone **6** (100 mg; 0.37 mmol), Ag_2O (425,1 mg; 1.8 mmol), anhydrous $MgSO_4$ (300 mg), 2-aminobenzothiazole **7** (61.5 mg; 0.41 mmol) and CH_2Cl_2 (15 mL). Eluent mixture: (20:18:2 petroleum ether/CH_2Cl_2/EtOAc). IR (KBr, cm^{-1}): 3423 (O-H); 1674 and 1649 (C=O); HRMS (M^+): m/z Calc. for $C_{22}H_{12}N_2O_3S_2$: 416.02893; found: 416.02929.

3.5. *13-Hydroxy-13-(2-thienyl)-7H-benzo[g]benzo[4,5]-3-fluorothiazolo[2,3-b]quinazolin-7,12-quinone* (**26**): was isolated (46.5 mg; 63%) as orange solid, m.p. 260.5-261 °C from 2-thienoyl-1,4-naphthohydroquinone **6** (45.1 mg; 0.17 mmol), Ag_2O (194.6 mg; 0.84 mmol), anhydrous $MgSO_4$ (300 mg), 2-amino-6-fluorobenzothiazole **10** (32 mg; 0.19 mmol) and CH_2Cl_2 (15 mL). Eluent mixture: (20:18:2 petroleum ether/CH_2Cl_2/EtOAc). IR (KBr, cm^{-1}): 3448 (O-H); 1676 and 1649 (C=O); HRMS (M^+): m/z Calc. for $C_{22}H_{11}FN_2O_3S_2$: 434.01951; found: 434.01923.

Note: *NMR data for compounds* ***24-26*** *are not reported due to it extremely low solubilities in the common solvents used in NMR spectroscopy.*
